# Recent advancements and perspectives of photoresponsive inorganic nanomaterials for cancer phototherapy and diagnosis

**DOI:** 10.1039/d5ra01153a

**Published:** 2025-05-12

**Authors:** Jiahui Chen, Hongyu Yu, Tingting Zheng, Xiuyun Zhang, Chen Chen, Peng Sun

**Affiliations:** a Department of Pharmacy, Shandong University of Traditional Chinese Medicine Jinan 250355 China ttz10_10@163.com zhangxiuyunsh@163.com; b Key Laboratory of New Material Research Institute, Department of Acupuncture-Moxibustion and Tuina, Shandong University of Traditional Chinese Medicine Jinan 250355 China 21129008@zju.edu.cn; c Innovation Research Institute of Chinese Medicine, Shandong University of Traditional Chinese Medicine Jinan 250355 China sunpeng@sdutcm.edu.cn

## Abstract

PTT (photothermal therapy)/PDT (photodynamic therapy) has unique advantages, such as its minimally invasive nature and clinical relative safety, and is considered a promising approach for cancer diagnosis and treatment. However, the therapeutic efficacy of phototherapy is often limited by the limited depth of light penetration and the low targeting of phototherapeutic agents. Recently, photoresponsive inorganic nanomaterials have flourished in the fields of PTT and PDT in cancer, providing a possible approach to enhance phototherapeutic potency. This review summarizes the recent research progress of common photoresponsive inorganic nanomaterials in the field of PTT and PDT and their diagnosis in cancer, involving noble metal nanoparticles, sulfide nanomaterials, oxide nanomaterials, and carbon nanomaterials. It focuses on the therapeutic and diagnostic performance of PTT and PDT of these inorganic nanomaterials and provides strategy improvements for expanding the drug delivery application of PTT/PDT. Finally, the future research and development of photoresponsive inorganic nanosystems for the treatment and diagnosis of PTT/PDT in cancer are discussed, and the possible opportunities and challenges are discussed.

## Abstract 2

The overview of various diagnostic and therapeutic modalities mediated by photoresponsive inorganic nanomaterials.

## Graphical abstract

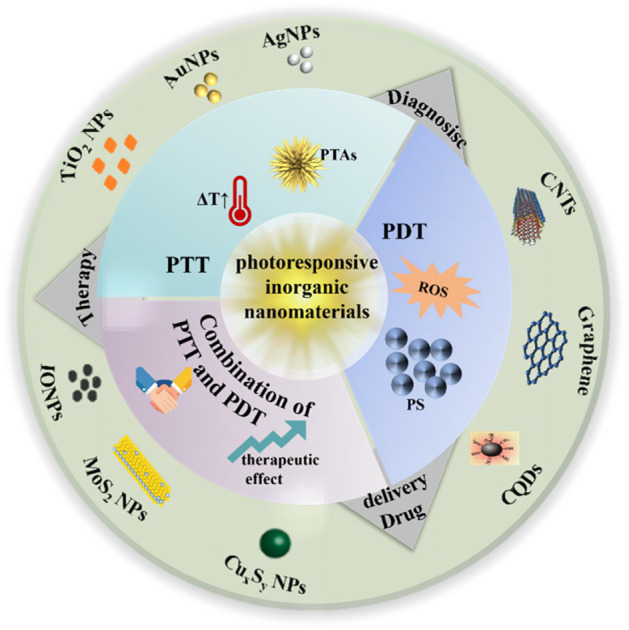

## 1. Introduction

Cancer is one of the leading causes of human death and one of the most serious public health problems worldwide. Early treatment of cancer is optimal. The only treatment is to kill cancer cells, but it is a challenge to selectively kill cancer cells without damaging healthy non-cancer cells. Traditional treatments include surgery, radiotherapy, chemotherapy, *etc.*, by which many cancers can be cured. However, some postoperative side effects may affect the patient's body and have a higher risk of tumor recurrence and metastasis. Therefore, as a non-invasive treatment that can be implemented at the cellular level with controllable time and space, phototherapy is an alternative or supplementary means of traditional treatment. Phototherapy is a local treatment based on light irradiation, which can be divided into PTT and PDT. Among them, PTT generates heat with photosensitizers (PS) with light absorption, causing irreversible cell damage and thus thermal ablation of tumors. PDT uses light-sensitive molecules to absorb light energy to produce cytotoxic reactive oxygen species (ROS), leading to apoptosis or necrosis of cancer cells.^1^ Both have little damage to healthy tissue, and can timely adjust the treatment scheme (such as irradiation area, time and injection dose) according to the physiological response and clinical needs of patients, which are two relatively effective new treatment methods at present.^2^ Additionally, timely diagnosis is crucial for offering a favorable chance for rehabilitation. In this sense, nanomaterials may open up new avenues for the diagnosis and treatment of cancer.

People have developed nanocarriers based on light response to low-energy photon irradiation, especially those located in the near-infrared (NIR) range, considering the poor tissue penetration and high phototoxicity of high-energy photons in ultraviolet/visible light. NIR light-triggered drug release systems are often achieved by using two-photon absorption and photon upconversion processes.^3^ Therefore, it is important to develop phototherapeutic diagnostic reagents with deep tissue penetration. Due to the dense and structurally intact microvascular endothelial gaps in normal tissues, macromolecules and lipid particles are not easy to penetrate the blood vessel wall, while the solid tumor tissue has abundant blood vessels, wide vascular wall gap, poor structural integrity, and lack of lymphatic reflux, resulting in retention of macromolecular substances and lipid particles, which is called the enhanced permeability and retention (EPR) effect of solid tumor tissue. Tumor blood vessels have good permeability, and the nano platform mainly penetrates into tumor tissue through the endothelial gaps of blood vessels. In phototherapy, inorganic nanotherapeutic platforms can have the EPR effects after systemic administration through passive targeting of the tumor site or by modifying the targeting molecules or high-affinity ligands. In general, solid tumors usually lack lymphatic vessels, so nanomaterials that enter the tumor gaps through external permeable absorption are retained. Meanwhile, nanoparticles have strong adhesion to the blood vessel wall of the tumor, and can also prolong the retention time and concentrate in the tumor. When the nanoplatform is enriched at the tumor site, the tumor can be treated with laser irradiation for PTT or PDT.^4^ Compared with inorganic materials, organic dyes such as methylene blue and cyanine blue have strong light-absorbing properties, but due to a series of reasons such as poor photostability, biotoxicity, fewer varieties, difficulties in synthesis, and higher costs, there are fewer practical applications. The photoresponsive inorganic nanomaterials used for PTT/PDT diagnosis and treatment generally have the following advantages: (1) large extinction coefficient, strong photostability, high photothermal conversion efficiency (PCE), and capability of producing ROS; (2) size and structure are easy to regulate and regulate the absorption peak through the size and structure;^1^ (3) easy to synthesize; (4) easy to modify molecules or functional groups on the surface of the material to reduce toxic side effects or improve the targeting and selectivity of treatment.

PDT and PTT based phototherapy have been identified as a tumor ablation modality for many cancer indications, in which PS and photothermal agents (PTAs) play a significant role in phototherapy. This paper aims to discuss the now-reported applications of various types of photoresponsive inorganic nanomaterials in phototherapy and the diagnosis of cancer. First, the mechanisms of PTT and PDT are introduced. Secondly, the commonly used treatment, diagnosis, and improvement strategies of PTT or PDT *in vitro* and *in vivo* for photoresponsive inorganic nanomaterials are highlighted. Finally, the challenges of photoresponsive inorganic nanomaterials in cancer phototherapy and potential opportunities for future development are discussed.

## 2. Introduction of PTT/PDT

### 2.1. Introduction of PTT

PTT is a technique that applies nanostructures and laser irradiation to tumor areas (or metastatic sites) to induce thermotherapy. In general, this treatment begins with the intravenous injection of nanomaterials, which can accumulate within the tumor through the EPR effects. PTT relies on PTAs with photothermal conversion ability to eliminate tumors at high temperature, which has the advantages of high precision and low toxicity. PTT has many advantages over traditional treatments, including minimally invasive and high specificity.^5^ The photothermal conversion mechanism of photoresponsive inorganic nanomaterials is the electron oscillation or lattice vibration after absorbing the light radiation, thus converting the light energy into thermal energy. Different types of materials have different electronic or bandgap structures and different responses to optical radiation, so the photothermal conversion mechanism is different. The PTT mechanism is roughly divided into three categories. The representative inorganic nanomaterials of each mechanism are noble metal nanomaterials (plasma local heating), semiconductor nanomaterials (non-radiative relaxation into heat), and carbon material (thermal vibration in molecule).

**Fig. 1:**
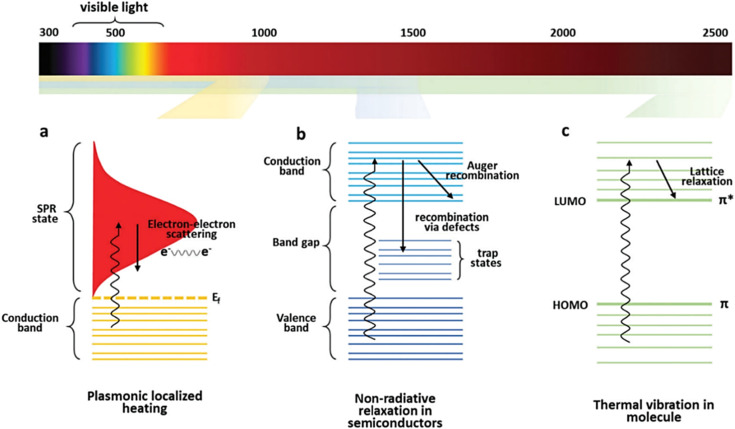
Different mechanisms of the photothermal effect with the corresponding light absorption range. (a) Plasmonic localized heating, (b) non-radiative relaxation in semicondutors and (c) thermal vibration in molecule. Reprinted with the permission from ref. 11. Copyright © Royal Society of Chemistry.

When noble metal nanomaterials are irradiated by light, the free electrons in the conduction band tend to produce collective oscillation. Local surface plasmon resonance (LSPR) refers to a resonant photon-induced coherent charge oscillation that occurs when the photon frequency matches the intrinsic frequency of electrons on the metal surface. This LSPR effect induces three consecutive phenomena: near-field enhancement, hot electron generation, and photothermal conversion.^6,7^ The plasmon photothermal effect is a relatively new research field that emerged in 2002 and is mainly utilized in medical applications, namely photothermal cancer therapy or drug delivery.^8,9^ The absorbed energy can then produce light scattering through radiative relaxation or nonradiative relaxation, which is converted to heat. The LSPR effect is strongly correlated with various factors such as particle morphology, size, composition, interparticle distance and dielectric properties.^1,10^ When metal nanoparticles are exposed to external irradiation at their resonant wavelengths, the plasmon-enhanced photothermal effect occurs. This phenomenon leads to the oscillation of the electron gas, where electrons are excited from occupied states to unoccupied states, generating hot electrons and resulting in a non-equilibrium charge distribution. These hot electrons decay either by radiative emission or by electron–electron interactions leading to carrier multiplication (Fig. 1a).

Semiconductor nanomaterials can be divided into two categories: one is semiconductors with defect structures such as copper sulfides and some transition metal oxides, and the other is semiconductors with intrinsic bandgap absorption, such as transition metal nitrides, carbides, and some chalcogenides. The defect structure of the former leads to the surface migration of the carriers and shows the collective oscillation of the free charge carriers (holes) in the valence band, thus producing the LSPR effect, which is different from the LSPR of noble metal from the collective oscillation of free electrons in the conduction band. Since the LSPR level of such semiconductors depends on the number of carriers and is independent of the material morphology, their photosensitivity is more stable. However, the photothermal conversion process of the latter depends on the intrinsic absorption band gap between the valence band and the conduction band. When the photon energy is greater than the band gap width, the electron absorbing the photon in the valence band jumps to the conduction band and leaves holes in the valence band to form an electron–hole pair. Subsequently, the stimulated electrons release energy back to the ground state by converting radiative relaxation to light energy or non-radiative relaxation to heat energy.^10^ Different types of semiconductor materials have different band gap widths between valence and conduction bands, and the range of light absorbed is also different^1^ (Fig. 1b). The photothermal effect is a result of the diffusion of light excitation and the temperature distribution of composite carriers in the material.

When carbon nanomaterials are irradiated with incident light that matches the possible electron transitions within the molecule, the electrons transition from the highest occupying molecular orbital (HOMO) to the lowest unoccupied molecular orbital (LUMO). Subsequently, the excited electrons are relaxed in the form of phonons, which initiates the vibration of the entire crystal lattice to achieve photothermal conversion. π-bonds are usually weaker than σ-bonds due to the lower bonding electron strength of π-bonds, and these electrons can jump from π-orbitals to π*-orbitals with lower energy excitation. In addition, the conjugated π bonds of carbon materials such as graphene and its derivatives also cause red shifts in the absorption spectrum. The higher the number of π-bonds, the smaller the band gap between HOMO and LUMO, and the easier it is for electrons to be excited.^11^ When the material is irradiated with light energy matching the possible electronic leaps within the molecule, the light-absorbing electrons are lifted from the ground state (HOMO) to higher-energy orbitals (LUMO), photothermal therapeutic mechanisms of different photoresponsive nanomaterials and their corresponding light-absorbing ranges as shown in (Fig. 1c). The excited electrons undergo relaxation *via* electron–phonon coupling. Consequently, the absorbed light energy is transferred from the excited electrons to the vibration modes of the entire atomic lattice, leading to a macroscopic rise in the temperature of the parent substance.

### 2.2. Introduction of PDT

PDT was discovered by Von Tappeiner and Jesionek in Germany more than a hundred years ago, and it wasn't until 1904 that it was discovered that the presence of oxygen was important for therapy, hence the name photodynamic.^12^ Currently, PDT is a highly successful non-invasive treatment for several skin disorders, such as psoriasis and cancer. There are three important elements to performing PDT, namely the presence of photosensitizer drugs, a light source, and oxygen. The interaction of these elements produces ROS, which play a key role in treatment.^13^ PDT can disrupt tumours through three possible modalities. The first mechanism is that the tumour is disrupted under light irradiation, generating ROS, and killing cancer cells through mechanisms such as apoptosis, autophagy and necrosis. In the second mechanism, PDT undermines tumour cells by attacking the vascular system and the tumour microenvironment. In the third mechanism, the tumour can be disrupted by generating or activating immune responses.^14^ The dual specificity of PDT is ensured by (i) enhanced PS accumulation in tumors and (ii) selective illumination of the diseased area.^15^

**Fig. 2:**
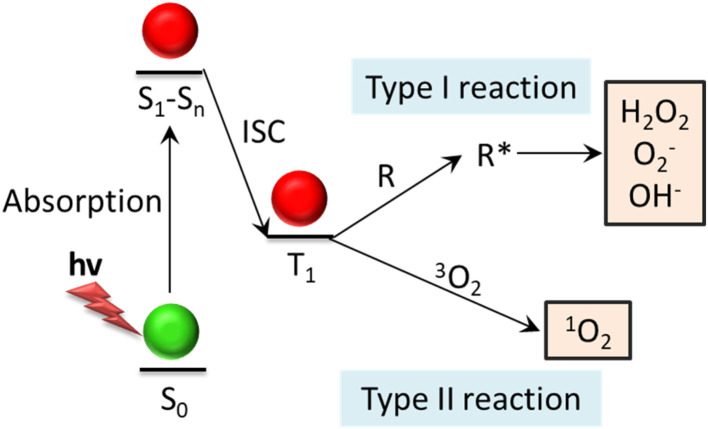
Demonstration of the mechanisms behind the type-I and type-II photodynamic reaction. The photosensitizer in the triplet state reacts with biological substrates (type I reaction) or surrounding oxygen (type II reaction) to produce highly toxic ROS. The ROS generated by type I reaction is active radicals, and ^1^O_2_ is produced by type II reaction. These ROS can oxidize tumor cell membranes, proteins, DNA, *etc.*, causing irreversible cellular damage. Abbreviation: ISC: intersystem crossing; R: biological substrate; R*: oxidized biological substrate.

Under laser irradiation at a specific wavelength, the PDT photosensitive material absorbing photons can transform from the ground state (S_0_) to the singly excited state (S_1_–S_*n*_), and then undergo intersystemic leaps to the triplet excited state (T_1_).^16,17^ A photosensitizer in the T_1_ state may undergo two different types of reactions, type I and type II, to generate highly cytotoxic ROS. In type I reactions, hydrogen atoms or electrons are transferred between the laser-sensitized molecule and the organic substrate to produce ROS such as H_2_O_2_, superoxide anion (O_2_^−^), hydroxyl radical (˙OH). In the type II reaction, the energy of the stimulated photosensitizer is transferred to the ground state molecular oxygen (^3^O_2_) to form a ROS called singlet oxygen (^1^O_2_).^15,18^ However, some research has indicated that nanomaterials like gold and silver nanoparticles can generate singlet oxygen when exposed to NIR light. Additionally, it has been shown that the morphology of these particles is mainly responsible for the generation of singlet oxygen. For example, singlet oxygen can be generated *via* photo-irradiation and sensitization of silver decahedrons and silver triangular nanoplates, but not by silver nanocubes and gold decahedrons.^19^ These ROS can then react rapidly with surrounding biological components such as the plasma membrane, peptides, proteins and nucleic acids, causing irreversible damage to target cells, thus killing tumor cells through apoptosis or necrosis.^20^ Moreover, PDT is also able to exert antitumor activity by damaging microvessels or inducing immune responses.^18^ The reaction mechanisms of type I and type II PDT are shown in Fig. 2.

PDT can be used safely together with standard antitumor therapies such as surgery, chemotherapy, and radiotherapy without reducing its clinical efficacy. In addition, PDT of skin tumors can achieve good cosmetic results, and the procedure is harmless to the connective tissue and does not cause scab. It is also worth mentioning that PDT can be performed in an outpatient setting, thereby reducing the cost of patient care. The construction of a wearable low-irradiated organic light-emitting diode improves the outpatient treatment of non-melanoma skin cancer.^15,21^

### 2.3. The combination of PTT and PDT

PDT, which results in localized chemical damage in the target lesions, and PTT, which results in localized thermal damage. The heat generated by PTT can increase blood flow, improve oxygen supply and enhance the PDT, whereas PDT can increase the sensitivity of tumour cells to PTT by interfering with the tumour microenvironment therapeutic effect.^22^ Additionally, ROS generated during PDT can disrupt heat-shock proteins (HSP), which are one of the reasons for the effects of PTT. The reciprocal collaboration among diverse therapeutic approaches may heighten the anti-tumor efficacy under low-dose PS or low-power light irradiation, thereby minimizing the potential toxicity to non-malignant tissues. Furthermore, the multimodal therapy integrating PTT and PDT holds great promise in countering multidrug resistance (MDR) and hypoxia-related resistance in cancer treatment.^23^

Alternatively, single laser-triggered simultaneous PTT and PDT, based on the use of a photothermal agent coupled with a photodynamic agent or a dual-modal photothermal and photodynamic agent, has been reported.^24,25^ This approach, which integrates PDT and PTT, simplifies the therapeutic process and leads to improved treatment outcomes in comparison with single-mode treatment in preclinical models. Nevertheless, it demands relatively high-power laser irradiation (≥1 W cm^−2^) for an extended period (>5 minutes) to trigger the synergistic PTT and PDT effects, and even for single-mode PTT activity, which has drawn attention. Hence, for the purpose of simplifying the treatment and averting laser-related toxicity, there is a need to develop methods that concurrently utilize PTT and PDT, namely, employing a single low-power NIR laser for short-term irradiation.^26^ Owing to reasons such as the insufficient PCE of PTT, it is challenging for PTT to achieve satisfactory performance in tumor treatment. The combined use of PTT with therapeutic modalities such as PDT can significantly enhance the tumor-killing capacity and has been extensively utilized in the development of therapeutic platforms. Copper sulfide nanoparticles (CuS NPs) as photothermal reagents possess the merits of low toxicity and straightforward synthesis. Hence, the combination of CuS NPs with PDT PS constitutes an effective strategy for establishing a PTT/PDT combined therapeutic platform. It has also been verified *in vivo* that the tumor suppression effect of the PDT/PTT combined treatment group is conspicuously greater than that of the single treatment groups.^27^ Upon irradiation with a single 808 nm laser, the NaGdF4 Er, Yb@NaGdF4:Nd@Cu(ii) boron imidazolate framework nanocomposites not only demonstrate outstanding photothermal conversion capacity but also generate cytotoxic ROS through *in situ* Fenton-like reactions and fluorescence resonance energy transfer. Significantly, these nanocomposites concurrently introduce remarkable anti-tumor efficacy *via* combined photothermal/photodynamic/chemodynamic therapy both *in vitro* and *in vivo*.^28^

Different photoresponsive inorganic nanomaterials, including metal nanomaterials,^29^ carbon-based nanomaterials,^30^ transition metal sulfides,^31^*etc.*, can produce a strong photothermal effect under the irradiation of light at specific wavelengths, and can also be modified to participate in PDT as PS through different preparation methods, such as nanoparticles, nanocomplexes, *etc.*, to achieve the synergy between PTT and PDT. The synergistic effect of PTT and PDT can be realized. This kind of combined therapy is expected to improve the therapeutic effect and reduce the side effects, which is an emerging direction in tumor therapy.

## 3. Application of photoresponsive inorganic nanomaterials in cancer diagnosis and treatment

Compared to organic nanoparticles, inorganic nanoparticles have significant advantages such as higher stability, ease of surface modification, suitability for specific targeting (specific ligands), size tunability and optical properties. They are also more useful in diagnostics or imaging. The intrinsic functionality and respective properties of photoresponsive inorganic nanoparticles have attracted the attention of the biomedical applied science community. The photoresponsive inorganic nanoplatforms discussed for phototherapy in cancer are mainly classified according chemical components into noble metal nanoparticles, sulfide, oxides, carbon nanomaterials and black phosphorus. The features of these reported photoresponsive inorganic nanomaterials for cancer phototherapy are shown in Table 1. By comparing the features in Table 1, the key properties, advantages, and drawbacks of these photoresponsive inorganic nanomaterials are summarised in Table 2.

**Table 1:** Representative photoresponsive inorganic nanomaterials for cancer PTT and PDT

Materials	Components	PCE (%)	ROS production (compared with the standard ROS kit)	Tumor model	Treatment parameters	Cell apoptosis *in vitro*/*in vivo* (%)	Ref.
Precious metal-based	Au-on-Au nanorods	67.2		HeLa tumor	PTT: 1060 nm laser (0.8 W cm^−2^, 10 min 55 °C), 0.3 mg kg^−1^ (injection in mice)	100	32
	g-C_3_N_4_/SnS_2_@Au	41		HepG2 tumor	PDT/PTT: 808 nm laser (0.75 W cm^−2^, 5 min 42.9 °C), 250 μg mL^−1^ (intratumorally inject)	80	33
	AuNC@Bi_2_Se_3_	42.1		4T1 tumor	PDT/PTT: 808 nm laser (1 W cm^−2^, 10 min 49.3 °C), 3 mg kg^−1^ (intravenous injection)	80	34
	Au@Pd bimetallic nanozyme	58.25		4T1 tumor	PDT/PTT: 1064 nm laser (0.5 W cm^−2^, 10 min 45.1 °C), 10 mg kg^−1^ intratumor administration	—	35
Transitional metal-based	Bi_2_S_3_/Ti_3_C_2_-TPP	42.13		U251 tumor	PDT/PTT: 808 nm laser (1 W cm^−2^, 10 min 51.9 °C, 20 mg kg^−1^ (intravenously inject)	100	36
	W_2_C NPs	46.8		S180 tumor	PDT/PTT: 1064 nm laser (0.8 W cm^−2^, 10 min 51.9 °C, 10 mg kg^−1^ (intravenously inject)	100	37
	Ti_3_C_2_T_*x*_ MXenes		1.31	4T1 tumor	PDT/PTT: 808 nm laser(1.3 W cm^−2^, 3 min 45 °C), 50 μg mL^−1^	—	38
	Nano-TiO_2_-coated MCNTs			HCT116 cells	PTT: 808 nm laser(1.5 W cm^−2^, 5 min), 100 μg mL^−1^ (tumor inject)	—	39
	Cu-SiNPs			4T1 tumor	PTT: 808 nm laser (1.5 W cm^−2^ 5 min), 64 mg Cu^2+^ mL^−1^ (intratumorally inject)	—	40
	MoS_2_–CuO heterostructures			CT26 tumor	PTT: 808 nm laser (1.5 W cm^−2^, 5 min) 5 mg mL^−1^ (intratumorally inject)	—	41
	WMoO_*x*_/CS	52.66		MCF-7 tumor	PTT: 1064 nm laser (0.7 W cm^−2^, 10 min > 50 °C), 20 mg kg^−1^ (intratumorally inject)	91.2	42
	MoS_2_@RP	36.56	0.496	786-O cells	PTT/PDT: 808 nm laser (2 W cm^−2^, 5 min 45.6 °C), 125 μg mL^−1^ (intratumorally inject)	—	43
	CuS@COF	63.4		4T1 tumor	PTT/PDT: 505 nm (50 mW cm^−2^) and 1064 nm laser (1 W cm^−2^, 50.8 °C), 100 μg mL^−1^ (tail vein injection)	85.1	27
	Aptamer@dox/GOD-MnO_2_-SiO_2_@HGNs-Fc@Ce6	41.3		HepG2 cells	PTT/PDT: 808 nm (1.6 W cm^−2^, 5 min) and 660 nm (0.2 W cm^−2^, 5 min 56 °C), 100 μg mL^−1^ (tail vein injection)	54.74/80	44
Carbon-based	nrGO-PEG/CSL			4T1 tumor	PTT: 808 nm laser (2 W cm^−2^, 5 min 70 °C), 2 mg kg^−1^ (intratumorally inject)	∼42	45
	BP-ester-C_60_	44.1		4T1 tumor	PTT: 1064 nm laser (1.5 W cm^−2^, 5 min 53.7 °C), (intravenous injection)	∼85	46
	rGOQD/IR820/MnO_2_/Q/CPP	54.2		U87 tumor	PTT/PDT: 808 nm laser (1 W cm^−2^, 5 min 51 °C), 0.5 mg mL^−1^ tail vein injection	—	47
	CQDs/ICG/DOX@LPs-FA	47.14		4T1 tumor	PTT: 808 nm laser (2 W cm^−2^, 6 min), DOX dose at 3 mg kg^−1^ and CQDs at 7.5 mg kg^−1^ (intravenous injection)	85.6	48
	rPPH@AZD	47.6		A549 cells	PTT/PDT: 660 nm + 808 nm laser (0.6 W cm^−2^, 2 min), (tail vein injection)	72.1	49
	3D CNT/MXene	82.9		Hela cells	PTT/PDT: 808 nm laser (1.5 W cm^−2^, 5 min 76.9 °C), 0.5 mg mL	59.4	50
	HMONs-rNGO@Fe_3_O_4_/MnO_*x*_@FA/DOX/TPP			Hela tumor	PTT/PDT/CMT: 808 nm laser (0.8 W cm^−2^, 10 min), (intratumorally inject)	—	51
BP	NBID (BP, ICG, DOX)	89.5		A549 cells	PTT/PDT: 808 nm laser (1 W cm^−2^, 10 min), 0.5 mg mL	84.24	52
	BP@ZIF-8	31.90		HCT-116 cells	PTT: 808 nm laser (1 W cm^−2^, 10 min 69.3 °C), 0.1 mg mL	73	53

**Table 2:** Overview of the different kinds of nanomaterials

Category	Typical materials	Key properties	Advantages	Drawbacks
Noble metals	AuNPs, AgNPs	Surface plasmon resonance effect, the biocompatibility is relatively high, the stability is high, the PCE is generally high, and the ROS generation is weak	High stability, adjustable optical properties inert and easy to modify	High cost, scarce resources, complex preparation process, less drug-loading capacity, limited photodynamic activity
Sulphides	MoS_2_, CuS	The biocompatibility is relatively low, the stability is general, the PCE is relatively high, and the ROS generation ability is relatively weak	Low cost, strong light absorption capacity, high heat conversion efficiency, high-specific surface area, abundant catalytically active sites	Poor solubility in the physiological environment, hazardous synthetic methods, limited surface functionalization strategies
Oxides	TiO_2_, Fe_3_O_4_	The biocompatibility is relatively good, the stability is relatively good, but the PCE is generally low, the ROS generation ability is good	Adjustable bandgap, efficient photoactivity, large surface area, low cost	Uncertain toxicity of degradation products, weak photoactivity to long-wavelength light, limited surface functionalization strategies
Carbon-based	CNTs, COQs	Biocompatibility is generally good, chemical stability is generally high, and most PCE are in the middle. ROS generation is generally lower than that of traditional PS	Light weight, versatile, chemical inertness, biocompatibility, large surface area to absorb light and molecules	Poor solubility in the physiological environment, limited photodynamic activity, limited size, and shape control
BP	BP@ZIF-8, NBID	Unique layered structure and adjustable band gap, biocompatibility is relatively good and degradable, but the stability is generally poor. PCE is close to noble metals and better than sulfides. ROS can be produced directly	Good biodegradability, the surface is easy to modify	More efficient coating technologies need to be developed, and long-term toxicity data are insufficient, sensitive to water and oxygen

### 3.1. Noble metal nanomaterials

Nanoparticles made from noble metals in the spotlight. Nanometals are bioinert (biologically inert), strongly light-absorbing, and can be tuned to improve absorption bands by adjusting their size, shape, and composition to modulate LSPR properties for clinical applications.^54^ Precious metal nanoparticles have good photostability and can be used with higher laser power and longer light exposure times.^6^ However, the LSPR resonance of metal materials is difficult to redshift to the NIR biology window, thus affecting the penetration depth of the incident light. At present, the absorption bands can only be tuned by fine tuning of microstructures (*e.g.* synthesizing nanorods, nanoshells, *etc.*), which has limited tunability and is a tedious process.^55^ In addition, the raw materials of noble metal nanoparticles are expensive, may be difficult to degrade in living organisms, and are susceptible to changes in morphology and properties under prolonged laser irradiation, which limit their development. To address these safety challenges and improve the efficacy of targeted therapies, stable biocompatible nanosystems were also explored.^56^

Gold is a widely used noble metal with low cytotoxicity, superior biostability and biocompatibility, and has been studied most in the biomedical field.^57^ Gold nanoparticles (AuNPs) can form many different forms of nanostructures such as gold nanoshells (AuNSs),^58^ gold nanorods (AuNRs),^59^ gold nanoclusters,^57,60^ gold nanostars^24^ and so on. AuNSs are composed of gold thin layer and dielectric core (such as SiO_2_), with good photostability. The photo-absorption of NIR region can be enhanced according to the appropriate core–shell ratio. They are the first classical nanoparticles used for PTT.^4,12^ New internal hollow-type AuNSs have been applied. Tang *et al.*^58^ explored for the first time he use of SN@AuNSs composed of mesoporous sandwiched SiO_2_ nanocores and thin gold shells for *in vivo* and *in vitro* PTT/chemotherapy combinations. SN@AuNSs were loaded with DOC up to 52%. SN@AuNSs has good thermal stability and mechanical stability, which leads to the unclear cumulative release of DOC triggered by NIR. *In vivo* and *in vitro* studies, it shows better synergistic anti-liver cancer effect than single chemotherapy or PTT. In addition, it was loaded with organic dyes for imaging and further diagnosis. The results show that SN@AuNSs is a multifunctional diagnosis and treatment system which can be used for PTT, drug delivery and cell imaging.

**Fig. 3:**
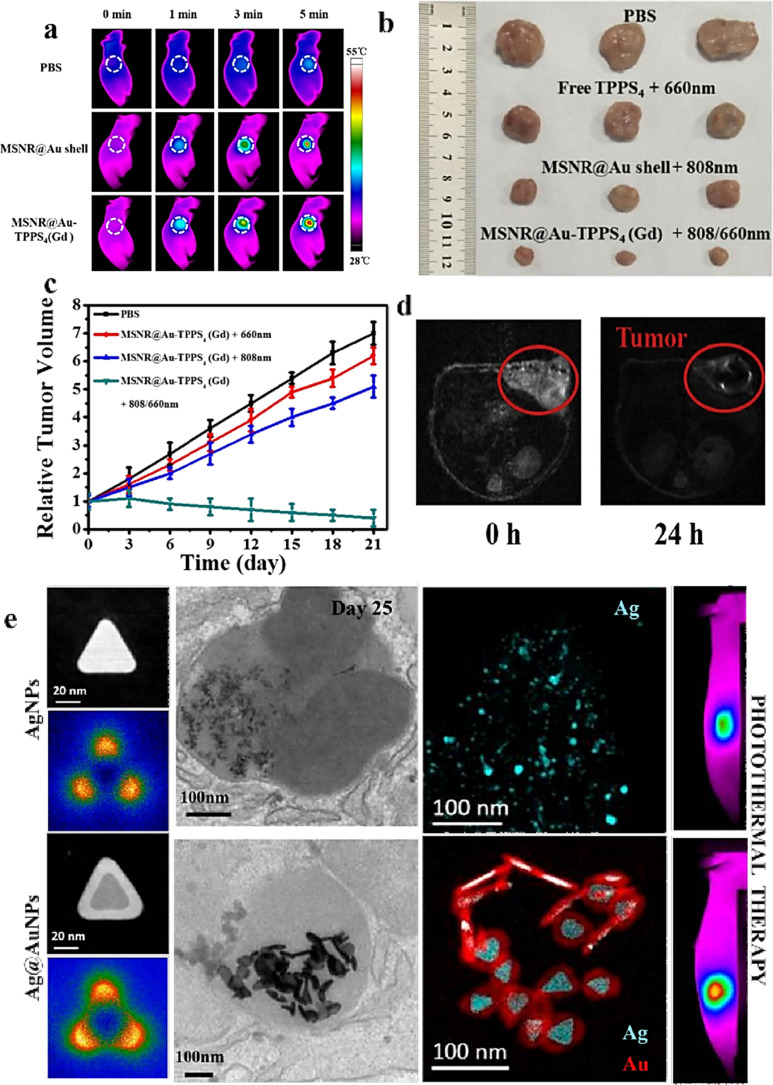
(a) Thermographic pictures of mice with 4T1 tumors with exposure of the 808 nm laser irradiation after injected with PBS, MSNR@Au shell and MSNR@Au-TPPS4(Gd). (b) Pictures of the tumors gathered from various groups at the termination of treatment. (c) Relative tumor volume curves of mice with 4T1 tumors in various treatment groups. Reprinted with the permission from ref. 59. Copyright © 2019 American Chemical Society. (d) *In vivo* T2-weighted MR images of a mice. Reprinted with the permission from ref. 64. Copyright © 2019 Amsterdam. (e) Annular dark field (ADF) STEM micrograph and EELS plasmon resonance maps of the corner (dipolar) mode of Ag and Ag@AuNPs. TEM images and EELS elemental maps of Ag and Ag@AuNPs in the cellular environment at days 25 after internalization in MSC stem cells. Reprinted with the permission from ref. 65. Copyright © 2019 American Chemical Society.

Gold nanorods (AuNRs) generally have the strongest plasma LSPR performance among gold nanoparticles with various structures. It has anisotropic shape and superior spectral bandwidth, and can be suitable for NIR treatment by adjusting the aspect ratio.^61^ Although AuNRs can be synthesized efficiently and on a large scale, the cetyltrimethylammonium bromide (CTAB) surfactant used in the synthesis may be cytotoxic, so it is difficult to meet the clinical application conditions. Therefore, it is necessary to change to other suitable reagents before *in vivo* application.^54^ In addition, the high uptake rate of AuNRs in the liver may affect their aggregation in tumors, thus requiring developing novel biocompatible vectors for their efficient delivery to tumors. Studies have shown that surface modification of PEG or polymeric nanorods can reduce the toxicity and liver accumulation of AuNRs.^62^ Quidant *et al.*^63^ studied the effects of different structural parameters of AuNRs (such as aspect ratio, length and molecular weight) on cytotoxicity *in vitro*, cell uptake and photothermal efficiency, and determined that AuNRs with a diameter of about 10 nm and with 800 nm-centered NIR absorption exhibit optimal cellular uptake and PTT efficiency during PTT treatment. Because AuNRs has the problems of small surface area and low drug loading, it is a good way to combine silica and gold to construct rod-shaped composite nanoparticles. Li *et al.*^59^ coated rod-shaped mesoporous silica with gold nanoshells, and then functionalized with ultra-small gadolinium (Gd) chelated supramolecular photosensitizer TPPS4 to prepare MSNR@Au-TPPS4 (Gd) nanoparticles. The composite nano-platform has good NIR absorption performance and excellent PCE (Fig. 3a). Anti-tumor experiments *in vivo* show that the PTT/PDT synergistic tumor treatment of the nano-platform is more effective than the single treatment mode, showing good anti-cancer effect (Fig. 3b and c).

**Fig. 4:**
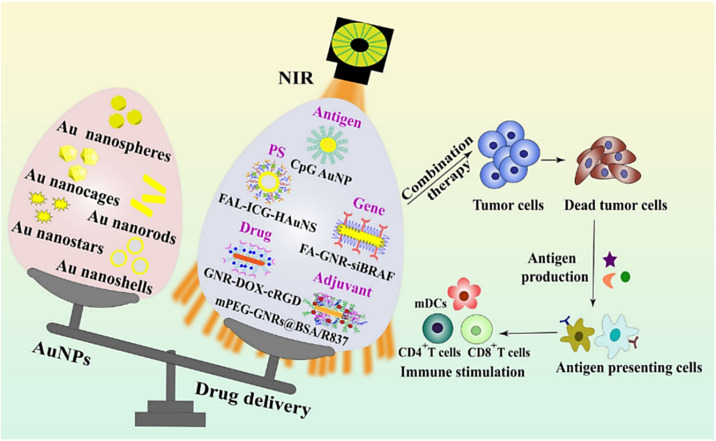
Schematic graph of different types of AuNPs and their applications in drug delivery to carry antigens, PS, genes, adjuvants, and chemotherapeutics. Reprinted with the permission from ref. 70. Copyright © BioMed Central. Abbreviations: BSA, bovine serum albumin; cRGD, cyclic arginine–glycine–aspartic acid; DOX, doxorubicin; FA, folic acid; FAL, ER-targeting pardaxin; GNR, gold nanorod; HAuNS, hollow gold nanosphere; ICG, indocyanine green; mDC, mature dendritic cells; mPEG, methoxypolyethylene glycol; siBRAF, small interfering RNA specifically silencing BRAF.

Compared with AuNPs, Ag Nanoparticles (AgNPs) have stronger LSPR performance, higher PCE, and can absorb NIR light energy. It is safe and effective to use AgNPs in cancer treatment and combine it with other materials. Shen *et al.*^64^ synthesized multifunctional Ag@Fe_3_O_4_@C core–shell nanocomposite with Ag as core and Fe_3_O_4_ and carbon as shell, where Ag was used as photothermal agent to enhance carbon and Fe_3_O_4_ as magnetic resonance imaging agent (Fig. 3d). The carbon shell modified surface functional groups and further enhanced excellent stability as well as tumor targeting. In many studies, Ag is often combined with Au to construct core–shell composite nanoparticles.^1^ For example, Wilhelm *et al.*^65^ used anisotropic silver nanosheets coated with gold shells to form composite nanoparticles (Ag@AuNPs). The nanoparticles combine the advantages of gold and silver, and the gold shell is used to protect the silver core from the harsh biological environment, so it still has excellent photothermal efficiency (Fig. 3e), which leads to the ablation of almost all tumors *in vivo*. Recently, AgNPs have attracted special interest in the field of nano-medicine, because some research groups have reported that these NPs can induce anti-tumor effects in tumor models *in vitro* and *in vivo*, which may be beneficial to many tumor treatment methods and diagnostic tools. AgNPs has high conductivity, catalytic activity and plasma properties, and can be used to improve the performance of biosensors, so it is a very attractive material for diagnosis. AuNPs can be functionalized to confer selectivity and targeting capability towards diseased tissues. Additionally, the integration of multiple imaging modalities, including computed tomography,^66,67^ surface-enhanced Raman scattering (SERS),^68^ and photoacoustic imaging^69^ has been explored in conjunction with PTT for enhanced cancer diagnosis and therapy (Fig. 4).

### 3.2. Metal sulphide nanoparticles

Recently, sulfide nanoparticles have attracted the attention of researchers for working as PTAs. Some features enhance the functionality of these nanomaterials, including: high NIR absorption, rapid human metabolism, high molar extinction coefficient, and high PCE.^71^ According to research, sulfide nanoparticles can absorb light and kill tumor cells by delivering energy to surrounding oxygen, producing highly reactive monooxygen or heat energy.^72^ In addition to the above advantages, the lower price of these nanoparticles compared to noble metals leads to expand their application in cancer PTT.^73^ Transition metal chalcogenides such as MoS_2_,^74^ WS_2_ (ref. 75) and Cu_*x*_S_*y*_ (ref. 76) have the structures of nanosheets and nanodots.

MoS_2_ nanosheets are promising new photosensitive materials. In recent years, MoS_2_ nanomaterials have received increasing attention in cancer diagnosis and treatment.^77^ Due to its two-dimensional (2D) nature, MoS_2_ exhibits the unique characteristics of 2D materials as well as semiconductor properties. These features render it highly promising for applications in tumor marker detection, tumor imaging and therapy. Firstly, MoS_2_ possesses a substantial specific surface area, enabling effective adsorption of various molecules such as nucleic acids, proteins, drugs, and fluorescent probes through covalent or non-covalent interactions to form MoS_2_ nanocomposites with radioactive, magnetic, and imaging functionalities. Furthermore, MoS_2_ nanocomposites also demonstrate outstanding controlled release by specifically responding to the tumor microenvironment.^78^ For example, Chou *et al.*^79^ synthesized a two-dimensional amphiphilic MoS_2_ sheet (ceMoS_2_) and investigated its effectiveness as a photothermal agent. The Morrison synthesis method enables the hydrophobic MoS_2_ to be dispersed in water and is also applicable to other transition metal disulfides. The absorbance of ceMoS_2_ under 800 nm NIR light irradiation was comparable to that of rGO, eight times larger than that of GO and two times larger than that of gold nanorods. *In vitro* experiments demonstrated that ceMoS_2_ produced good cell disruption. Liu *et al.*^80^ synthesized MoS_2_ nanosheets and functionalized them with thionic acid modified polyethylene glycol (LA-PEG). The ultra-high specific surface area of the fabricated MoS_2_-PEG allowed efficient loading of drug molecules. After loading DOX, MoS_2_-PEG exerted significant photothermal/chemotherapeutic synergistic therapeutic activity *in vitro*, and showed excellent tumor inhibition after *in vivo* injection. Although Mo is an essential trace element and this study also showed that MoS_2_-PEG had no significant cytotoxicity, more detailed studies are needed to understand its potential long-term toxicity. This effort reveals for the first time the great potential of transition metal disulfides for use as novel 2D drug-carrying nanosystems in combined tumor therapy.

**Fig. 5:**
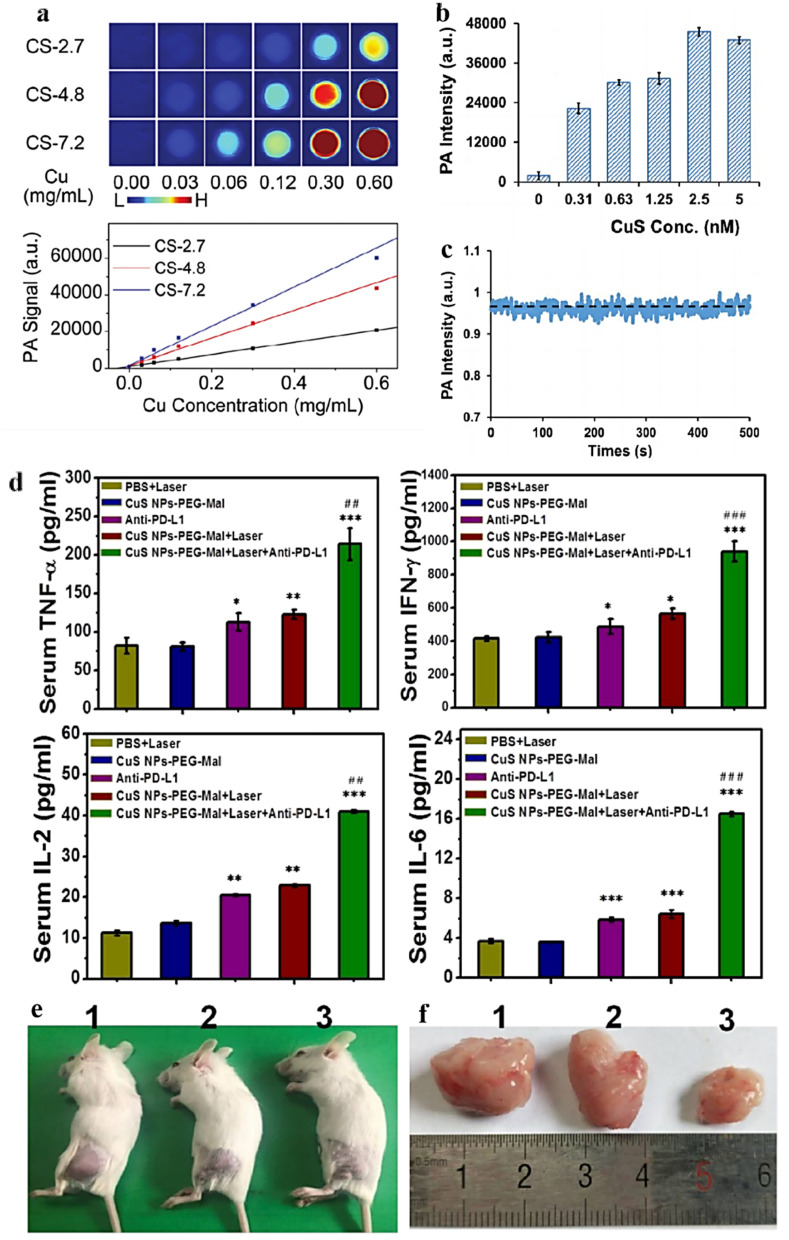
(a) *In vitro* photoacoustic imaging (upper frame) and the correspondingly quantified PA signal (lower frame) of aqueous solutions containing differently sized PEGylated copper sulfide nanoparticles. Reprinted with the permission from ref. 81. Copyright © 2019 RSC Pub. (b) Photoacoustic scan of CuS nanodisk. (c) The signal stability of photoacoustic intensity of CuS nanodisk. Reprinted with the permission from ref. 82. Copyright © 2019 VCH Verlagsgesellschaft. (d) ELISA analysis of cytokine levels in sera isolated from mice after different treatments. (e) Representative photos of the 4T1 tumor-bearing mice after treatment for 12 days. (f) Photographs of the collected tumor tissues for each treatment group. (1) PBS control group, (2) CuS NPs-PEG-Mal group, (3) CuS NPs-PEG-Mal adsorbing protein antigens group. Reprinted with the permission from ref. 83. Copyright © 2019 American Chemical Society.

Copper sulfide nanoparticles (CuS NPs) is an emerging nanoplatform with dual diagnostic and therapeutic applications. Ding *et al.*^81^ devised monodisperse and size-controllable polyethylene glycolized CuS nanoparticles (3–7 nm) *via* an aqueous-phase synthesis route, which resulted in CuS nanoparticles that were highly stabilized in a colloidal state (Fig. 5a). *In vitro* and *in vivo* analysis of the contrast-enhancing effect of the nanoplatforms revealed that the effect was directly proportional to the particle size. The nanosystem (<5 nm) developed in this study exhibited favorable tumor imaging performance in HeLa cells. Wang *et al.* prepared CuS nanodiscs by solvent-based synthesis and surface modified them with polyethylene glycol methyl ether thiol. The CuS nanodiscs exhibited a strong surface-enhanced resonance effect in the NIR region. Size tunability was obtained at lower concentrations, and the small size of the CuS nanodisks made them viable imaging nanoplatforms^82^ (Fig. 5b and c). Copper sulfide compounds (Cu_*x*_S_*y*_) are promising new PTAs due to their low cost and toxicity and NIR absorption peaks.^2^ Pellegrino *et al.*^76^ prepared plasma copper (Cu_2−*x*_S) sulfide nanocrystals (Cu_2-*x*_S NCs) and reported for the first time their PTT/PDT synergy under NIR light irradiation. To combine high PCE with the ability to absorb breast tumor protein antigens. Wang *et al.*^83^ developed surface-modified copper sulfide nanoparticles and modified the same with maleimide PEG. In a triple-negative breast cancer tumor model, the nanoparticle-mediated PTT was capable of inducing alterations in the immune microenvironment and blocking the anti-PD-L1 checkpoint (Fig. 5d). The research findings indicated that in the 4T1 tumor model, with the elevation of inflammatory cytokine levels, the quantity of CD8+ T cells increased significantly, thereby suppressing the growth of primary and distant tumors (Fig. 5e and f). Overall, the surface-modified CuS nanoparticles in this study served two functions: (1) as a photothermal coupling agent to ablate tumors, and (2) to absorb released antigens during PTT and transfer them to dendritic cells. Thus, this study provides a simple and effective therapeutic strategy for tumor.

In addition to the above-mentioned and widely studied sulfide, there are also other semiconductor sulfide nanomaterials used in PTT/PDT and cancer diagnosis. Cadmium sulfide (CdS) nanoparticles are also typical semiconductor nanoparticles, which can be used in PDT to generate reactive oxygen species, used to kill cancer cells. Subsequent investigations have indicated that the pristine CdS nanoparticles might inflict damage on accumulated tissues. Through this research effort, we have discovered that the modified CdS nanoparticles could potentially serve as effective imaging agents for the detection of cancer and other diseases.^84^ Lead sulfide nanoparticles show potential application in PTT by absorbing light energy to generate thermal energy for killing cancer cells. Preliminary experimental results by Sun *et al.*^85^ show that lead sulfide quantum dot (PbS-QD) bioconjugates are valuable for future *in vivo* tissue imaging as a NIR contrast agent for targeted molecular imaging with extended emission wavelengths beyond 1000 nm. Nickel sulfide nanomaterials also have potential applications in cancer diagnostics. Although nickel sulfide is relatively new in the field of cancer diagnostics, its soft ferromagnetic characteristics make it a promising diagnostic tool that shows great potential as a multifunctional therapeutic agent for biomedical applications.^86^

### 3.3. Metal oxide nanoparticles

The application of metal oxides in magnetic resonance imaging (MRI) and tumor therapy is a research hotspot in the field of nanomedicine. MRI is superior to other imaging methods in structural and molecular state imaging. Its unique physical and chemical properties (such as magnetism, catalytic activity, biocompatibility, *etc.*) provide new strategies for disease diagnosis and treatment. Currently, among the mainstream noninvasive diagnostic methods in the clinic, MRI shows advantages for radiation-free, full-view assessment, and real time imaging.

**Fig. 6:**
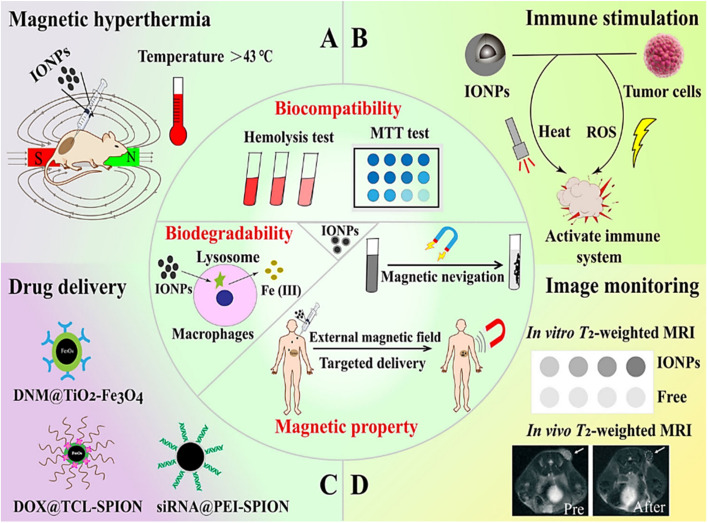
Summary of the typical characteristics of iron oxide nanoparticles (IONPs) and their major functions in cancer therapy. The inside of the circle illustrates the biodegradable, biocompatible, and magnetic properties of IONPs. The outside of the circle indicates various applications of IONPs in biomedical field. (A) Magnetic hyperthermia effect for anti-tumor therapy. (B) Activation of the immune system by IONPs combined with PTT and PDT. (C) IONPs as carriers to deliver therapeutic agents. (D) The application of IONPs in concentration-dependent T2-weighted MRI. Abbreviations: DNM, daunomycin; PEI, polyethylenimine; ROS, reactive oxygen species; SPION, super paramagnetic iron oxide nanoparticle; TCL, thermally cross-linked. Reprinted with the permission from ref. 70. Copyright © BioMed Central.

Iron oxide nanoparticles (IONPs) are one of the most promising MRI contrast agent precursors for cancer diagnosis.^87^ Furthermore, IONPs are capable of converting the magnetic energy within an alternating magnetic field into thermal energy, effectively ablating cancer cells under high-temperature circumstances. There are different magnetic particles with dual magnetic function and NIR absorption that can convert light into heat, where IONP is well suited for PTT. The efficiency of IONP in the biomedical field is enhanced by properties such as biodegradability, biocompatibility, ease of synthesis, ease of conditioning, and the possibility of using it as an MRI contrast agent in the clinic.^88^ These nanoparticles are able to absorb various kinds of light (from visible to NIR light) and convert light energy into heat energy.^89^ The IONP acts as a resonant object that increases energy conversion and causes intense cell death. In addition, commonly used NIR waves increase penetration into tissue depths. Along the endocytosis pathway, IONPs are metabolized to elemental iron by lysosomal and endosomal hydrolases. Consequently, these nanoparticles can be absorbed by the organism and maintain iron homeostasis while generating the slightest side effects. Furthermore, another benefit of employing these magnetic nanoparticles in cancer PTT lies in that through the application of an external magnetic field, the active and passive targeting of IONPs towards tumor sites can be strengthened. As tumor-specific targeting ligands can be coupled to these nanoparticles, the accuracy of treatment can be further enhanced.^90^ Alternating magnetic fields (AMF) can induce bipolar relaxation of IONPs and generate magnetic hyperthermia. Magnetic heat is capable of specifically destroying tumor tissues, reducing damage to healthy tissues, and the local temperature can be precisely controlled through adjusting the concentration of nanoparticles at the tumor site and the intensity of AMF.^91^ Consequently, IONPs possess extensive application prospects in cancer PTT. Superparamagnetic iron oxide nanoparticles (SPIONs), consisting of small-sized iron oxide crystals (such as magnetite Fe_3_O_4_ or magnetic red mite γ-Fe_2_O_3_), are capable of attaining colloidal stability in aqueous media *via* surface modification. Nanosize and magnetic field sensitivity make SPION unique. The combination of these nanoparticles with different molecules enables their widespread application^92^ (Fig. 6). Iron oxide nanoparticles are magnetic and photosensitive and can be used in magnetic and thermal therapy combined with PTT to achieve more precise therapeutic positioning.

With advances in nanotechnology, fluorescent dyes or magnetic resonance contrast agents have been successfully labeled as titanium dioxide nanoparticles, nanotubes, or nanoprobes for cellular imaging by fluorescence analysis or MRI.^93^ Furthermore, through the utilization of highly sensitive B-TiO_2_-based SERS biological probes, rapid and precise diagnosis of MCF-7 drug-resistant (MCF-7/ADR) breast cancer cells can be accomplished. The results provide a new approach for the design of novel semiconductor nanomaterials, with an efficient photoinduced charge transfer (PICT) transition and significant surface-enhanced Raman scattering (SERS) sensitivity, thus broadening the application of the semiconductor-based SERS platform in precision diagnostics and treatment of cancer.^94^ At the same time titanium dioxide nanoparticles have been widely studied in PDT, which can be used to destroy cancer cells by stimulating the production of reactive oxygen species.^95^ The photodynamic activity of titanium dioxide is based on its ability to generate ROS under UV irradiation, which leads to lipid peroxidation in its vicinity resulting in cellular damage. The hybridized ZnPc@TiO_2_ nanostructures designed by Flak *et al.* have a high potential to act as selective biological imaging agent, in addition to their photodynamic activity and drug delivery capabilities.^96^ A novel tie-shaped molybdenum dioxide (MoO_2_) nanoparticle was successfully synthesized by Liu *et al.* These nanobows have a strong LSPR effect in the visible to NIR region and exhibit ultra-high chemical stability. Under NIR laser irradiation, they showed excellent PTT effect, significantly inhibited the viability of cancer cells *in vitro*, and effectively destroyed the growth of tumor tissue *in vivo*.^97^ Molybdenum dioxide nanoparticles synthesized by Wang *et al.*^98^*via* hydrothermal synthesis have a high photothermal conversion rate and excellent photothermal stability. The results of the cytotoxicity test demonstrate that this nanomaterial poses no notable toxicity to normal cells. Meanwhile, it exhibits a remarkable photothermal killing effect on liver cancer cells, thus promising great potential as nanomedicines in cancer therapy.^99^ Another important biomedical semiconductor material is tin dioxide (SnO_2_). It exhibits moderate antimicrobial, antioxidant, and cytotoxic properties.^100^ Throughout the treatment process, imaging tracking is indispensable for tumor diagnosis in order to improve the accuracy of treatment and reduce damage to normal tissues.^101,102^ Among several imaging modalities, PA imaging has the advantages of high spatial resolution and deep tissue penetration, using light-guided ultrasound to detect tumor location.^103–106^ Thus, PA imaging may have a prominent role in image-induced cancer therapy. In addition, the construction of hyaluronic acid on SnO_2−*x*_ could be targeted by interacting with cells through the CD44 protein receptor overexpressed on the cell surface, thus the composite SnO_2−*x*_@SiO_2_-HA nanomaterials realized PA image-guided PDT. *In vivo* and *in vitro* anti-tumor experiments showed that SnO_2−*x*_@SiO_2_-HA had a significant inhibitory effect on tumor growth, and even ablated completely.^107^

In general, oxide nanomaterials have shown broad application prospects in the field of PTT and diagnosis, and can provide effective means for the early detection, precise localization and individualized treatment of tumors.

### 3.4. Carbon-based nanomaterials

The main carbon nanomaterials that have been developed for cancer diagnosis and treatment are carbon nanotubes (CNTs),^108^ carbon quantum dots (CQDs)^109^ and graphene.^110^ These materials are characterized by ultra-high specific surface area, large drug-carrying capacity, strong light-absorbing capacity, high PCE, *etc.* In addition, they have excellent photoluminescence performance, both photothermal and photodynamic dual high-efficiency NIR phototherapeutic functions, which can be used for intracellular imaging, high-efficiency molecular loading and biocoupling, and have been widely used as a new type of nanocarriers for drug and gene delivery.

**Fig. 7:**
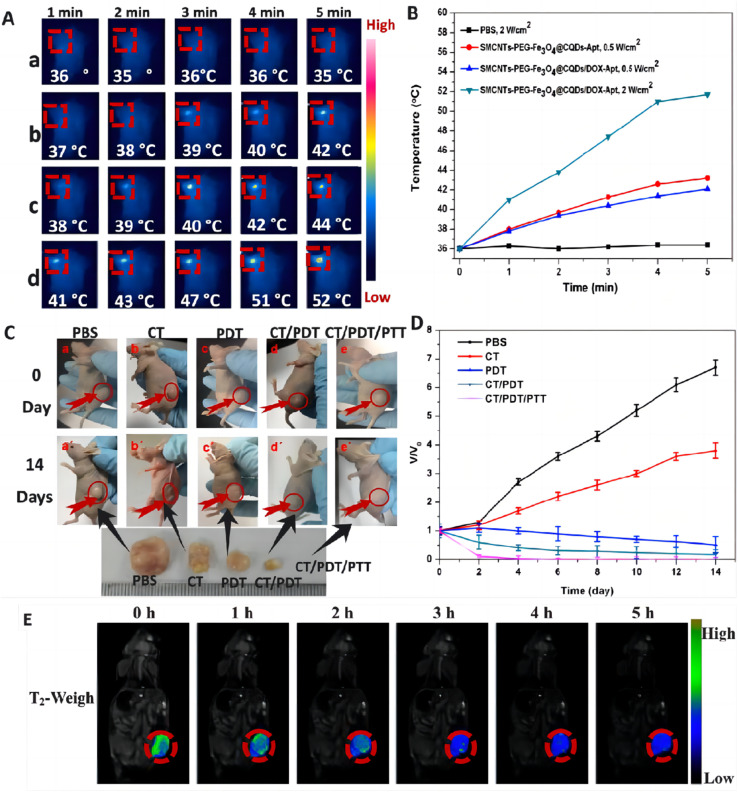
(A) IR thermal images of HeLa tumor-bearing mice incubated with various media: (a) PBS, (b) 100 μg mL^−1^ of SWCNTs-PEG-Fe_3_O_4_@CQDs-Apt + laser, (c) 100 μg mL^−1^ of SWCNTs-PEG-Fe_3_O_4_@CQDs/DOX-Apt + laser and (d) 100 μg mL^−1^ of SWCNTs-PEG-Fe_3_O_4_@CQDs/DOX-Apt + laser. (B) Heating curve of the five laser-irradiated groups. (C) Representative photographs of tumor-bearing mice after different treatments. (D) Time-dependent tumor growth curves observed after different treatments. (E) *In vivo* T_2_ weight MR images and color-mapped images of mice bearing tumor after intravenous injection of SWCNTs-PEG-Fe_3_O_4_@CQDs at different timed intervals. Reprinted with the permission from ref. 112. Copyright © 2018 Elsevier.

CNTs are carbon nanomaterials with unique structure and excellent properties, consisting of single or multi-layer graphene sheets curled into a cylindrical, long and hollow structure, which are now widely used in a variety of industries. CNTs are suitable for use as drug carriers, can be loaded with a variety of targeted ligands and drugs, and can be modified appropriately to reduce the toxicity of the drug, so as to make it biocompatible.^61,111^ They can be used as photothermal converters for PTT, which kills cancer cells by absorbing light energy and converting it into heat. In addition, CNTs can be used as PS for PDT, destroying cancer cells by generating substances such as ROS. Their unique physicochemical properties have made them a popular tool for cancer diagnosis and treatment. Shen *et al.*^112^ prepared SWNTs-PEG-Fe_3_O_4_@CQDs nanocomposite material, which has photodynamic and photothermal effects under 808 nm laser irradiation, can also load drugs, and release drugs with pH/NIR photothermal response. The fabricated SWCNTs-PEG-Fe_3_O_4_@CQDS/DOX-Apt can be used for targeted dual-modality fluorescence/magnetic resonance (MR) imaging and PTT/PDT/chemotherapy combination therapy (Fig. 7).

CQDs have emerged as materials with extensive application potential in the biomedical domain owing to their fluorescence characteristics, water solubility, biocompatibility, low toxicity, small size, facile modification, low-cost large-scale production, and multifunctional combination with diverse nanoparticles. Consequently, CQDs have emerged as the preferred material for numerous biomedical applications, such as drug nanocarriers, gene therapy vectors, PS, and antibacterial molecules. Furthermore, their potential in multifunctional diagnostic platforms, cellular and bacterial bioimaging, and therapeutic diagnostic nanomedicine has been thoroughly validated.^113^ CQDs are novel carbon-based nanomaterials with excellent optical properties and biocompatibility. They can be used as fluorescent probes for cancer diagnosis, which can be realized by specific binding to biomolecules for the detection of cancer cells. In addition, CQDs can be used as PS for PDT. Therefore, CQDs show good application value in the field of nanomedicine (especially tumor diagnosis and therapy).^114,115^ Mustafa *et al.* have devised a highly sensitive fluorescent probe for the early detection of sarcosine, which is regarded as a potential biomarker of prostate cancer. The sensor is founded on cobalt-doped fluorescent carbon quantum dots (Co-CD) and adopts a FRET-based photoluminescence sensing platform. Blue luminescent CQDs were synthesized by hydrothermal method using Delonix regia tree pod shells. The lower limit of detection (LOD) for copper was 2.4 μM with a linear range of 0–10 μM. The limit of detection for sarcosine in phosphate buffer solution (PBS, pH 7.4) was 1.54 μM with a linear range of 0–10 μM. Importantly, the sensor demonstrated its applicability in clinical analysis by detecting sarcosine in human urine. In conclusion, this experimentally designed rapid and highly sensitive sensor provides a new method for the detection of sarcosine in real samples, which is helpful for the early diagnosis of prostate cancer.^116^

**Fig. 8:**
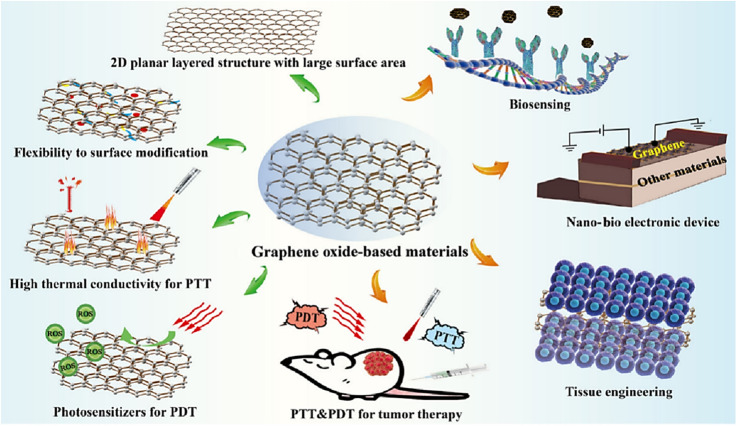
Schematic demonstration of the important characteristics of graphene oxide-based nanomaterials and their biomedical applications. Reprinted with the permission from ref. 70. Copyright © BioMed Central.

Graphene is a two-dimensional material composed of a single layer of carbon atoms, which also has important applications in cancer treatment. Graphene-based nanomaterials (GBN), with the advantages of target selectivity, easy functionalization, chemical sensitization, and high drug loading capacity, are potential drug carriers with potential use in breast cancer diagnosis and treatment.^117^ Graphene can be used as a photothermal converter to convert light energy into heat energy for PTT. It can also be used to prepare optical probes for cancer diagnosis. Various inorganic nanoparticles can be grown on the surface of graphene nanoparticles to obtain functional graphene nanocomposites with interesting optical and magnetic properties, which can be used for multimodal imaging and imaging-guided cancer therapy^118^ (Fig. 8). Liu *et al.*^119^ used branched PEG-functionalized nanographene oxide (GO-PEG) is loaded with Chlorin e6 (Ce6) *via* π–π stacking for PTT-enhanced PDT. GO-induced PTT under 808 nm laser did not result in cell death but triggered a mild localized heating, which could improve the cell membrane permeability, and dramatically facilitated the intracellular uptake of Ce6, which enhanced the efficacy of Ce6 for PDT. This study reveals the promising potential of graphene in cancer combination therapy.

Due to the wide use of graphene, the current research on graphene analogues is also booming. Barsoum's team^120^ discovered Ti_3_C_2_ in 2011 and named it transition metal carbon/nitride (MXenes). MXenes is a new type of 2D nanosheet composed of transition metal carbon, nitride and carbonitride layer. The molecular formula is M_*n*+1_X_*n*_ (M for early transition metals (Ti, V, Cr, Nb, Ta, Hf, Zr, *etc.*), and X for carbon or nitrogen). After Ti_3_C_2_ was developed, various types of MXenes have also been explored in the biomedical field. This kind of material has the advantages of large specific surface area, surface hydrophilicity, good biocompatibility, strong optical absorption, wide spectral range, and adjustable LSPR effect, but there are still many obstacles to further application, such as the higher toxicity of fluorine-containing reagents used in the synthesis.^1^ Their development in the direction of treating tumors is constrained by the limitations of existing design methodologies. These methodologies lack control over the size and distribution of tumors. Moreover, their PDT effect is poor. To address this unmet medical need, Gao *et al.*^50^ designed a simple strategy that processes MXene with carbon nanotube (CNT) into a three-dimensional (3D) honeycomb structure having anti aggregation capacity was established. Under light irradiation at 650 and 808 nm, 3D CNT/MXene microspheres could efficiently produce ROS. The results of the study suggest that this structure can be used for phototherapy of tumors, bacteria, and viruses for diseases, such as PTT, PDT, and multimodal synergistic therapy. 3D CNT/MXene with efficient PDT and PTT effects are a promising multifunctional cancer therapy platform.

**Fig. 9:**
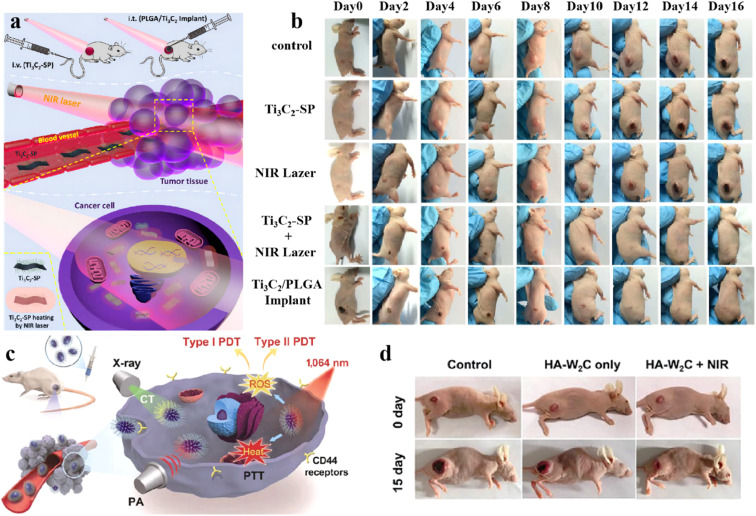
(a) Schematic diagram of intravenous and intratumoral injections. (b) Digital photos of 4T1 tumor bearing mice in the groups of the control, Ti_3_C_2_SP only, NIR laser only and Ti_3_C_2_SP + NIR laser taken and PLGA/Ti_3_C_2_ implant taken during 16 days' period after different treatments. Reprinted with the permission from ref. 122. Copyright © 2019 American Chemical Society. (c) Schematic illustration of application of W_2_C NPs for 1064 nm activated tumor dual-type PDT, PTT, and PA/X-ray CT dual-modal bioimaging. (d)Representative photos of tumor-bearing mice before and after 15 days of treatment (control: treatment with PBS). Reprinted with the permission from ref. 37. Copyright © 2019 Tsinghua University Press.

In order to achieve the synergistic effect of diagnosis and treatment, Wang *et al.*^121^ prepared a multifunctional nanoplatform composed of Ti_3_C_2_ nanosheets, aggregation-induced emission active PS and upconversion nanoparticles (UCNPs). The platform ingeniously integrates the intrinsic and remarkable of each component in a single formulation, ultimately enabling fluorescence imaging/photoacoustic imaging/photothermal imaging three-modal imaging-guided synergistic PDT/PTT. Due to the hydroxyl groups on the surface of Ti_3_C_2_ nanosheets, they aggregate immediately in physiological environment. To solve this problem, Shi *et al.*^122^ used soybean phospholipid (SP) to modify the ultrathin Ti_3_C_2_ nanosheets to improve their physiological stability. Due to the LSPR effect of Ti_3_C_2_, it showed strong absorption and PCE under 808 nm irradiation. Efficient *in vivo* photothermal ablation was achieved after intravenous injection of SP-modified Ti_3_C_2_ and intratumoral injection of PLGA/Ti_3_C_2_-SP, respectively (Fig. 9a). The PLGA/Ti_3_C_2_ implant ensures that the implant components does not leak into the systemic circulation and has excellent biological safety (Fig. 9b). The results show the great potential of Ti_3_C_2_ nanosheets for the treatment of PTT in cancer. Yang *et al.*^37^ developed W_2_C nanoparticles, which produced strong near infrared-II (NIR-II) light absorption after HA functionalization, and produced both type I (·OH) and type II (^1^O_2_) ROS using only 1064 nm laser irradiation (Fig. 9c). HA-W_2_C has good PCE, causing significant destruction of tumors in PDT/PTT synergistic therapy *in vitro* and *in vivo* (Fig. 9d), and can be used for the efficient treatment of deep and hypoxic tumors and imaging diagnosis.

### 3.5. Black phosphorus

In 2014, ultrathin black phosphorus nanosheets (BP NSs), also called phosphorene, were first exfoliated.^123^ BP NSs are emerging 2D layered photosensitizer with high-precision optical response and band gap that can vary with thickness, and its wrinkled morphology makes it large in terms of specific surface area. In addition, phosphorus is an indispensable element in the human body, which makes BP nanosheets biocompatible and biodegradable, and has broad prospects for clinical application.

**Fig. 10:**
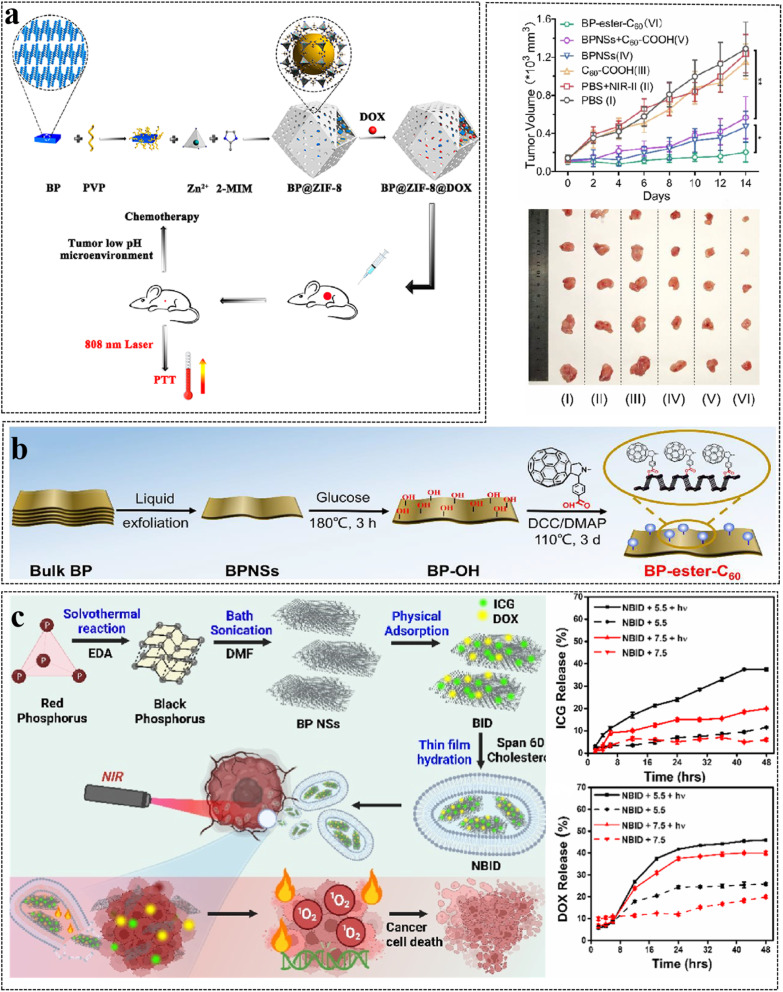
(a) BP@ZIF-8@DOX and Applications in Chemo-Photothermal Combined therapy. Reproduced from ref. 53 with permission from ACS, copyright 2021. (b) The preparation process and treatment effect of BP-ester-C60. Reproduced from ref. 46 with permission from ACS, copyright 2023. (c) NBID facilitating drug release for chemo-PTT of cancers. Reproduced from ref. 52 with permission from ACS, copyright 2024.

Meanwhile BP NSs is easily oxidized by oxygen and water under ambient environment, which affects its further popularization and application. Therefore, reliable BP passivation techniques for biomedical applications is urgently needed. Several passivation techniques of BP nanosheet including covalent aryl diazonium functionalization, capping layer protection, and ligand surface coordination. Wang *et al.*^53^ established a simple and applicable biomedical passivation strategy by encapsulating BP nanosheet into zeolitic imidazole framework-8 (ZIF-8). Due to the distinctive porous structure and pH sensitivity of ZIF-8, BP@ZIF-8 displays a high loading amount for DOX and pH responsive drug delivery property, making it an ideal candidate for multimodal therapy of tumors (Fig. 10a). Xie *et al.*^46^ developed a novel fullerene covalent passivation method *via* covalently grafting C60 onto a few-layer BPNS surface (named BP-ester-C60), which was applied in NIR-II PTT for the first time. In view of the *in vivo* antitumor NIR-II PTT research studies, BP-ester-C60 exhibits a higher tumor volume inhibition rate of ∼85% than the pristine BPNSs (∼58%) (Fig. 10b).Fullerene covalent passivation is effective in boosting the ambient stability and the PTT performance of BP, smoothing the way for the applications of BP in cancer treatments. The current research trend is gradually shifting to the development of biocompatible, controlled, and stable carrier systems. Vankayala *et al.*^52^ report NIR responsive BP NSs integrated niosomes to mediate chemo-phototherapy of cancers. Niosome-coated black phosphorus nanosheets loaded with ICG and DOX (NBID) exhibit very high drug loading efficiency (>90%). Upon laser irradiation, NBIDs offer excellent photothermal stability, elevated temperature, and generation of ROS. According to the literature, this is the first literature example in which the combination of two FDA-approved drugs, ICG and DOX, is coloaded onto the black phosphorus nanosheets and then integrated into niosomes to mediate chemo-PTT (Fig. 10c).

To achieve the synergistic effect of diagnosis and treatment, Lin *et al.*^124^ first used a single step conventional electrostatic attraction method to combine BP NSs with IONPs and gold nanoparticles, designing a multifunctional composite diagnostic and therapeutic agent BPs@Au@Fe_3_O_4_ based on the novel photosensitizer BP. BPs@Au@Fe_3_O_4_ integrates the PDT/PTT effects of BP, the photothermal effect of gold nanoparticles, and the targeting and MRI-guided capabilities of Fe_3_O_4_ nanoparticles, with high absorption bandwidth and physiological stability. Compared to BP, BPs@Au and BPs@Fe_3_O_4_, BPs@Au@Fe_3_O_4_ exhibits higher PTT and PDT efficiencies under 650 nm light exposure and performs excellently in combined PTT/PDT both *in vitro* and *in vivo*. In the future, it can be further utilized in efficient integrated cancer diagnosis and therapy strategies. Additionally, BP quantum dots (BPQDs) are ultra-small derivatives of BP NSs. The PDT/PTT anti-tumor combined therapy using BPQDs has been successfully validated. For example, Li *et al.*^125^ reported a multifunctional nano-diagnostic and therapeutic platform based on BPQDs. They prepared BPQDs with an average diameter of 2.5 nm and modified them with PEG chains. The small size of BPQDs allows for rapid clearance from the body. *In vitro* and *in vivo* studies have confirmed that under combined irradiation with 625 nm and 808 nm lasers, BPQDs exhibit excellent PDT/PTT combined anti-cancer effects, surpassing the efficacy of single-treatment modes. However, the PDT/PTT combined therapy using BPQDs still requires stimulation from two different wavelengths of laser. Therefore, it is necessary to study how single wavelength NIR light can simultaneously trigger PDT/PTT effect in the clinical application of BPQDs.

## 4. Improvement strategy

### 4.1. Material-based improvements

Solutions to PTT deficiencies: synthesis of new materials with higher PCE and NIR absorption capacity, enhanced thermal production capacity of nanomaterials under NIR laser irradiation, synthetization of materials that are responsive to the pH or oxygen gradient of the tumor microenvironment, and improvement of PTT strategies for nanomaterial mediation, including optimization of the size of nano-material: after being irradiated, nano-materials will scatter and absorb light, Wang *et al.*^126^ confirmed that while MoS_2_ nanosheets with a particle size of 100 nm exhibit higher NIR absorption than MoS_2_ nanosheets with a particle size of 80 nm, the latter can produce greater temperature variations under laser radiation due to their increased thermal efficiency. Tang *et al.*^127^ produced Pd-PVP nanoparticles in sizes of 41 and 4.4 nm and verified that the smallest nanomaterials have the highest thermal conversion efficiency. To accomplish effective and selective PTT under laser irradiation, nanomaterials must accumulate within tumor tissues and be internalized by cancer cells. This process is affected by factors such as the size, shape, surface charge, corona composition of the nanomaterials, and the presence of surface-targeting ligands.^128^ In conclusion, the development of new strategies or the optimization of the combination of existing strategies will undoubtedly significantly advance the application progress of nanomaterials in cancer phototherapy and diagnosis.

### 4.2. Delivery-based improvements

A highly promising platform for the treatment of solid tumors is capable of effectively delivering and releasing anti-cancer nanomedicines within tumor cells. Nevertheless, multiple biological barriers, especially those related to the tumor microenvironment, hinder the arrival of these therapeutic agents at the target cells. In this paper, we report a continuous pH and reduction-responsive polymer and gold nanorod (AuNR) core–shell assembly system that overcomes these obstacles through a two-stage size reduction and disassembly of the nanoplatform.^129^ There are two ways to improve the selectivity of PDT therapy to enhance the damage to cancer tissue without affecting the normal tissue around it: first, by packaging the therapeutic dye into the nanoparticles, and second, by using a radioprotector to intercept the photodynamic response in normal tissues, while allowing PDT to destroy the tumor. When photodynamic therapeutic drugs are encapsulated in stable and biocompatible silica nanoparticles, not only can the water solubility, stability and delivery efficiency of the drugs be significantly enhanced, but also their photodynamic therapeutic efficacy can be strengthened. Thiols can quench most reactive oxygen species, including singlet oxygen, superoxides, and lipid peroxides. In clinical studies, we found that thiols decreased the skin phototoxic response caused by endogenous porphyrins.^12,57^ Furthermore, thiols and thiol phosphates can cross the blood-eye barrier and prevent photo-oxidative damage of the retina and lens, with thiols remaining outside the tumor, thus protecting normal tissue from damage by reactive oxygen species. In addition, they are potentially biotoxic because they are difficult to degrade *in vivo*. It has been suggested that the toxicity of CNTs is related to preparation, shape and functionalization, which may affect their tumor targeting and therapeutic efficiency.^61,62^ Surfactants and inorganic residues used to increase the solubility of CNTs may induce immune and inflammatory responses. Therefore, systematic studies on issues such as the biocompatibility of CNTs are needed.

To the best of our knowledge, PDT-mediated monoclinic oxygen species are highly reactive molecules that are not naturally eliminated in mammalian cells, which provides an attractive and challenging opportunity to further enhance the efficacy of anticancer therapies. Drug delivery systems (DDSs) based on nanomaterials have shown a promise for cancer therapy. In this regard, the photoresponsive drug delivery system (DDS) that uses light as an external stimulus can achieve precise spatiotemporal control of drug release at the target location. NIR photoresponsive DDS has been developed. These systems can achieve on-demand release of nanomaterials in tumor tissues of living animals through photothermal, photodynamic, and photoconversion mechanisms, and synergize with phototherapy to significantly enhance the therapeutic effect. Cancer phototherapy can be achieved by improving the size and surface modification of nanomaterials to improve the PCE and increase the imaging and diagnostic functions; modifying the targeting molecules to achieve active, passive and triggered targeting, and further realizing the precise release of nanomaterials for accurate treatment; PDT with encapsulated chemotherapy nanoplatforms in hypoxic environments and constructing photoresponsive drug delivery nanosystems to improve the therapeutic effects and reduce the side effects of chemotherapy drugs.

### 4.3. Light-source-based improvements

In numerous circumstances, the outcome of combined therapy is not merely the superimposition of the individual effects of each treatment but rather demonstrates a synergistic action. PTT not only is capable of directly eliminating cancer cells but also strengthens other therapeutic approaches by enhancing drug efficiency, facilitating drug release, regulating the tumor microenvironment, inducing the release of tumor-specific antigens, or influencing other biologically relevant responses. It is worth mentioning that conventional PTT kills tumor cells by irradiating the tumor tissue by increasing the temperature to more than 48 °C to apply thermal ablation. However, thermal ablation during PTT can result in harmful damage to surrounding normal tissue, post-treatment inflammation, rapid metastasis or other side effects due to short-term massive release of tumor cell contents. To circumvent this limitation, mild-temperature photothermal therapy (MTPTT) was introduced instead of PTT, thermal therapy at moderate temperatures (41.8–45 °C) is able to produce a therapeutic effect on malignant cells in a hypoxic environment while causing minimal damage to surrounding healthy tissue.^115^ For example, using appropriate laser dose, determining the optimal treatment time after the administration of PTAs, improving the PCE of PTAs, and improving the delivery efficiency of PTAs in tumors by adjusting the shape, size and surface chemistry of the nanoparticles as well as tumor microenvironment. In addition, PTT in combination with other therapies has also shown improved therapeutic outcomes.^128,130^

The availability of different PS and different light sources makes PDT a complex process. In most cases, it needs to be optimized for each patient, which may not be an ideal function for standard therapy. Off-target photosensitization is a clinical problem since none of the clinically approved PSs are tumor-specific. Finally, most antitumor therapies currently in use are immunosuppressive, which may worsen treatment outcomes. In the case of carbon materials, for example, there are still many potential barriers to their clinical application, including poor dispersion and stability in water and harsh preparation and functionalization conditions,^2^ poor bioavailability affects their tumor targeting and penetration.^62^

**Fig. 11:**
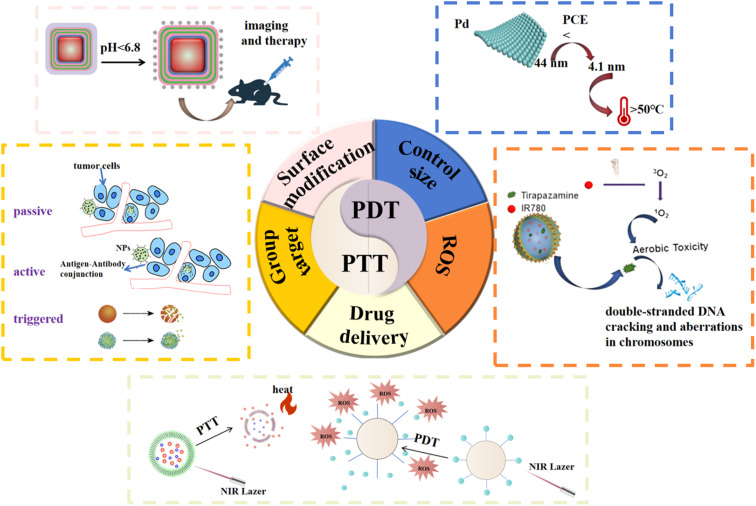
Strategies for improving cancer PTT/PDT.

There are still some challenges in PTT treatment of cancer. Firstly, one of the principal predicaments that PTT confronts is the restricted penetration depth of light, which might lead to incomplete ablation of tumors beyond the irradiation scope. In addition to this, other drawbacks include the relatively low efficiency of delivery of PTAs in tumors, overheating of the tumor region and unnecessary damage to normal tissues, and the overexpression of heat shock proteins in some cancers leading to the development of drug resistance to PTT.^115^ We report here new therapeutic strategies to improve the effectiveness of PDT while limiting side effects on normal tissues. For example, searching for new PS, developing new light sources, targeting PDT-induced cytoprotective mechanisms in tumors, establishing more effective PDT combination therapy, enhancing PDT-induced immune response, and new nanotechnology-based techniques. The different strategies are used to enhance the thermal therapy effects of nanomaterials mediated so far, namely those that improve nanomaterial accumulation in tumors (*e.g.* by changing the coronary structure or functionalizing through targeted adhesives), increasing the inherent ability of nano-materials to generate light heat (for example, by synthesizing new nanomaterials or assembling nanostructures), or by optimizing laser-related parameters used in radiation processes (for instance, by modulating radiation wavelengths) (Fig. 11).

## 5. Summary and outlook

In summary, various types of photoresponsive inorganic nanomaterials have become an important class of candidates in cancer diagnosis and treatment, and a large number of reports have been made on the remarkable achievement *in vivo* and *in vitro* experiments. The U.S. Food and Drug Administration (FDA) has approved various inorganic nanomaterials for use in the biomedical field, including drug delivery, diagnostic imaging, antimicrobial therapy, and medical device coatings. For instance, Fe_2_O_3_/Fe_3_O_4_ are used for contrast agents in MRI and anemia treatment (iron supplements), AgNPs for antimicrobial dressings, catheter coatings, and wound care, silica nanoparticles for fluorescence labeling of tumor boundaries during surgery. Therefore, it is essential to further explore these applications. However, there are still many challenges and opportunities in promoting these results for further clinical use: (1) there is an urgent need to develop biodegradable inorganic photoresponsive nanomaterials with adequate studies on *in vivo* pharmacokinetics and potential toxicity. (2) Since the synthesis of some multifunctional composite nanoplatforms can be complex, the design of multifunctional therapeutic platforms with diagnostic and therapeutic functions using a single nanostructure is of unique significance. (3) The development of phototherapy depends on the penetration of light into tissues. Bhatia *et al.*^131^ irradiated deep tumors by implanting a biocompatible NIR light source to achieve PTT, and new methods and techniques, new devices, *etc.*, that can improve penetration will continue to drive the change in oncology phototherapy. (4) Since the distribution of photoresponsive inorganic nanomaterials in tumors varies greatly over time, it is particularly important to determine the time at which the concentration of PS at the tumor site peaks for treatment. (5) For PDT, the therapeutic efficacy of PS capable of type II response only is limited for hypoxic tumors, and the development of PS capable of generating only type I ROS or both type I or type II ROS is a valuable research direction. (6) Combining PTT or PDT with other therapeutic modalities can make full use of the advantages of each modality to produce additional or even synergistic effects, which can improve the cure rate of tumors, reduce the toxic side effects, and reduce tumor recurrence and metastasis. For example, in PDT/PTT synergistic therapy, heat released from PTT can significantly improve intratumor blood flow and enhance oxygen supply, thereby enhancing cellular uptake of PDT reagents.^132^ (7) Small-sized nanosystems are rapidly cleared by the kidneys but result in a shortened circulating half-life, rapidly decreasing blood concentrations and reduced tumor uptake, compromising therapeutic efficiency.^60^ Therefore, the characteristics of the tumor microenvironment can be exploited to develop inorganic nanoplatforms that can be rapidly excreted from normal organs after systemic administration while effectively retained at the tumor site.

From the current trend of cancer diagnosis and treatment, the ultimate challenge for researchers is how to achieve non-invasive diagnosis and cure of tumors while avoiding toxic side effects. As the research and development of various types of photoresponsive inorganic nanomaterials are receiving more and more attention, photoresponsive inorganic nanomaterials-based PTT/PDT will provide safer and more effective options for cancer diagnosis and treatment, and they will have a profound impact on the field of cancer therapy. Regardless, these nano-carriers have the potential to make significant strides towards diagnosis and treatment of different diseases due to their unique qualities such as good spatial resolution, controllable drug release and active targeting for efficient and target-specific delivery of the encapsulated agent. The preparation and application of photoresponsive inorganic nanoplatforms in cancer PTT and PDT are highly explorable and will remain one of the major research directions in biomedicine in the future. We also expect that the development of photoresponsive inorganic nanomaterial-based nanotechnology could bring more opportunities for integrated therapeutic and diagnostic approaches. Future studies can benefit from the discussions and perspectives provided in this review.

## Data availability

No primary research results, software or code have been included and no new data were generated or analysed as part of this review.

## Author contributions

Jiahui Chen and Hongyu Yu searched references and wrote the manuscript. Peng Sun and Chen Chen were in charge of drawing figures and sorting tables. Tingting Zheng and Xiuyun Zhang revised the manuscript. All of the authors have read and approved the final manuscript.

## Conflicts of interest

The authors declare no conflicts of interest.
